# Characterization of integrative and conjugative elements carrying antibiotic resistance genes of *Streptococcus suis* isolated in China

**DOI:** 10.3389/fmicb.2022.1074844

**Published:** 2022-12-22

**Authors:** Jianping Wang, Kexin Qi, Xuemei Bai, Zongfu Wu, Weiming Kang, Pujun Liang, Han Zheng, Jianguo Xu

**Affiliations:** ^1^State Key Laboratory of Infectious Disease Prevention and Control, National Institute for Communicable Disease Control and Prevention, Chinese Center for Disease Control and Prevention, Beijing, China; ^2^OIE Reference Lab for Swine Streptococcosis, MOE Joint International Research Laboratory of Animal Health and Food Safety, College of Veterinary Medicine, Nanjing Agricultural University, Nanjing, China; ^3^Nanxishan Hospital of Guangxi Zhuang Autonomous Region, Guilin, China

**Keywords:** *Streptococcus suis*, antibiotic resistance, ICE, IME, *snf2* gene

## Abstract

*Streptococcus suis*, an emerging zoonotic pathogen, is important reservoirs of antibiotic resistance genes that play critical roles in the horizontal transfer of corresponding resistances. In the present study, 656 antibiotic resistance (AR) genes were detected in 154 of 155 genomes of *S. suis* strains isolated from the nasopharynx of slaughtered pigs and the lungs of diseased pigs in China. The AR genes were clustered into 11 categories, consisting of tetracycline, macrolides, lincosamide, streptogramin, aminoglycoside, trimethoprim, amphenicols, nucleoside, quinupristin/dalfopristin, glycopeptide, and oxazolidinones resistance genes. In order to investigate the transmission patterns of the AR genes, AR genes-associated the mobile genetic elements (MGEs) were extracted and investigated. Twenty ICEs, one defective ICE, one tandem ICE, and ten prophages were found, which mainly carried tetracycline, macrolides/lincosamides/streptogramin (MLS), and aminoglycosides resistance genes. Three types of DNA cargo with AR genes were integrated into specific sites of ICEs: integrative mobilizable elements (IMEs), *cis*-IMEs (CIMEs), and transposon Tn*916*. Obvious differences in AR gene categories were found among the three cargo types. IMEs mainly harbored tetracycline and MLS resistance genes. CIMEs mainly carried aminoglycoside resistance genes, while transposon Tn*916* carried only the *tet* (M) gene. Nearly all AR genes in ICEs were carried by IMEs and CIMEs. IMEs were prevalent and were also detected in additional 29 *S. suis* genomes. The horizontal transfer of IMEs and CIMEs may play critical role in ICE evolution and AR gene transmission in the *S. suis* population. Our findings provide novel insights into the transmission patterns of AR genes and the evolutionary mechanisms of ICEs in *S. suis*.

## 1. Introduction

*Streptococcus suis* is a major swine pathogen that causes septicemia, meningitis, and a variety of other diseases. It is emerging as a significant zoonotic agent responsible for the largest outbreak in China and increasing human meningitis worldwide (Ye et al., 2006; Gottschalk et al., 2007). High rates of resistance to tetracycline, macrolide, lincosamide, and aminoglycoside have been reported in both human and pig isolates of *S. suis* since 2000 (Wisselink et al., 2006; Ye et al., 2008; Zhang et al., 2008; Princivalli et al., 2009; Palmieri et al., 2011b). Furthermore, *S. suis* is likely an important reservoir of antibiotic resistance (AR) genes that may promote the interspecies horizontal transfer of these resistances (Palmieri et al., 2011b). A better understanding of the dissemination mechanism of AR genes is essential for monitoring and controlling drug resistance. Previous studies have revealed that mobile genetic elements (MGEs) play significant roles in the horizontal transfer of AR genes in *Streptococcus* (Puopolo et al., 2007; Chancey et al., 2015; Davies et al., 2015; Kambarev et al., 2018). Integrative conjugative elements (ICEs), prophages and tandem ICE-prophages located at different insertion sites were common MGEs described in *S. suis* (Uruen et al., 2022). Integrative and conjugative elements (ICEs) comprise the majority of MGEs and are critical contributors to the acquisition and distribution of AR genes in *S. suis* (Huang et al., 2016a), in which harbored tetracycline, macrolide, and lincomycin resistance genes (Li et al., 2011; Palmieri et al., 2011b; Marini et al., 2015; Athey et al., 2016; Huang et al., 2016a,b; Zheng et al., 2018; Libante et al., 2019; Pan et al., 2019; Shang et al., 2019; Zhu et al., 2019; Dechene-Tempier et al., 2021; Lunha et al., 2022; Yang et al., 2022). On the contrary, tetracycline, macrolide, lincomycin, nucleoside, and aminoglycoside resistance genes associated tandem ICE-prophages and prophage were often reported in *S. suis* clinical strains (Palmieri et al., 2011b; Huang et al., 2016a; Wang et al., 2019). In previous studies, tetracycline, macrolide, lincomycin, amphenicols, oxazolidinones, and aminoglycoside resistance genes associated prophages were common in *S. suis* strains (Wang et al., 2021; Uruen et al., 2022). Recently, the *optrA*-carrying prophage ΦSC181 was also reported in *S. suis* strain (Shang et al., 2019). In addition, IMEs mainly carrying tetracycline and macrolide resistance genes appeared to be more widespread than ICEs in *S. suis* and became major vehicles of AR genes in *S. suis* (Libante et al., 2019; Liang et al., 2021).

In order to thoroughly investigate the acquisition and dissemination mechanisms of AR genes, we genetically characterized AR genes-associated ICEs extracted from 155 genomes of *S. suis* strains isolated between 2006 and 2016 in China. Considering the fact that integrative mobilizable elements (IMEs) were widely spread in *S. suis* (Libante et al., 2019; Liang et al., 2021), the prevalence of IMEs with AR gens in the 155 genomes was also investigated.

## 2. Materials and methods

### 2.1. Bacterial strains, chromosomal DNA preparation, sequencing, and bioinformatic analysis

A total of 155 draft genomes of *S. suis* strains isolated from the nasopharynx of slaughtered pigs and the lungs of diseased pigs were included in this study (Supplementary Table S1). All strains were confirmed to belong to *S. suis* using 16S rRNA sequencing. They were sequenced on the Illumina platform as described in our previous studies (Chen et al., 2013; Qiu et al., 2016; Zheng et al., 2017). Sequences were assembled using SOAPdenovo (release 1.04), genes were predicted using Glimmer, and gene orthologs were determined using OrthoMCL.

The serotype of the tested strains was determined by capsular gene typing (Qiu et al., 2016; Zheng et al., 2017) and agglutination testing (serum provided by Statens Serum Institute, Copenhagen, Denmark; Supplementary Table S1). Multilocus sequence typing (MLST) were performed as described previously (King et al., 2002; Chen et al., 2013; Supplementary Table S1). The *rplL* gene, *rum* gene, *mutT* gene, luciferase-like monooxygenase gene, and ADP ribose pyrophosphatase gene are major insertion hot spots for the integration of MGEs in *S. suis* (Huang et al., 2016a; Libante et al., 2019). IMEs were usually inserted in *snf2* and *ppi* loci. Downstream sequences of these genes were therefore analyzed. Inserted sequences with no gap were further annotated using the bioinformatic methods described in a previous study (Zheng et al., 2018). Genes were annotated with the Artemis program. The Clusters of Orthologous Groups (COG) and Pfam protein motif databases were used to search for conserved protein domains. For ICE identification, signature proteins integrase, relaxase, and VirB4 were typed using the database from a previous study (Huang et al., 2016a). The clade to which each protein belonged was determined based on protein identification and coverage of greater than 90%. If all three proteins were present, the element was considered an ICE. Elements that lacked any of the three proteins were considered defective ICEs (DICEs). Search strategies and the definitions of IMEs and CIMEs have been described in a previous study (Coluzzi et al., 2017). Antibiotic resistance analysis was performed by searching the Antibiotic Resistance Genes Database (ARDB), the Comprehensive Antibiotic Resistance Database (CARD), and ResFinder of Center for Genomic Epidemiology^1^. A gene was only regarded as a resistance gene homolog if its protein showed at least 80% amino acid identity to a known resistance protein across 80% of the protein’s length (Hu et al., 2013; Table 1). Sequence comparisons were performed using the BLASTn program (E-value cutoff 1e^−10^) and visualized using an in-house perl script (available on line:^2^).

**Table 1:** Characteristics of ICEs, DICE and tandem ICE identified in the present study.

Name of ICE	Site of integration	VirB4	Integrase	Relaxase	Group	G + C content of ICE	Size of ICE (bp)	G + C content of IME	G + C content of CIME	Integrase of IME	Relaxase of IME	Insertion site of IME	Insertion site of CIME
ICE*Ssu*14ND70	*rplL*	IIIb	Vb	III	1	37.21	74,238	/	37.78	/	/	/	inside of *snf2* gene
ICE*Ssu*14ND1	*rum*	IIIb	III	II	4	38.66	69,332	47.65	/	Serine3	pfam01076	inside of *snf2* gene	/
ICE*Ssu*YS3	*rplL*	IIIb	Vb	IVa	2	38.69	62,097	46.18	/	Serine3	pfam01076	inside of *snf2* gene	/
ICE*Ssu*YS10	*rplL*	IIIb	Vb	IVa	2	38.13	70,132	43.64	/	Serine3	pfam01076	inside of snf2 gene	/
ICE*Ssu*YS17	*rplL*	IIIb	Vb	II	3	38.52	73,713	/	41.74	/	/	/	inside of *snf2* gene
Tandem ICE*Ssu*YS19	*rplL*	II + IIIa	IV + Va	I + IVa	/	38.38	112,763	46.63	/	Serine3	pfam01076	inside of *snf2* gene	/
ICE*Ssu*YS31	*rplL*	IIIb	Vb	IVa	2	38.08	76,286	44.09	/	Serine3	pfam01076	inside of *snf2* gene	/
ICE*Ssu*YS34	*rplL*	IIIb	Vb	IVa	2	38.61	64,136	44.71	/	Serine3	pfam01076	inside of *snf2* gene	/
ICE*Ssu*YS64	*rum*	IIIb	III	II	4	37.92	66,108	44.02	/	Serine3	pfam01076	inside of *snf2* gene	/
ICE*Ssu*YS66	*rplL*	IIIb	Vb	IVa	2	37.78	72,025	39.69	/	Serine3	pfam01076	3′ side of Peptidylprolyl isomerase	/
ICE*Ssu*YS108	*rplL*	IIIb	Vb	III	1	39.3	52,425	/	/	/	/	/	inside of *snf2* gene
ICE*Ssu*YS146	*rplL*	IIIb	Vb	IVb	5	37.33	77,439	/	/	/	/	/	/
ICE*Ssu*YS156	*rplL*	IIIb	Vb	III	1	37.73	61,170	/	38.08	/	/	/	inside of *snf2* gene
ICE*Ssu*YS162	*rplL*	IIIb	Vb	IVa	2	38.34	75,619	44.56	/	Serine3	pfam01076	inside of *snf2* gene	/
ICE*Ssu*YS165	*rplL*	IIIb	Vb	III	1	37.73	61,188	/	38.08	/	/	/	inside of *snf2* gene
DICE*Ssu*YS172	*rplL*	/	Vb	IVa	/	38.37	49,357	44.5	/	Serine3	pfam01076	inside of *snf2* gene	/
ICE*Ssu*YS196	*rplL*	IIIb	Vb	III	1	37.85	67,475	/	38.15	/	/	/	inside of *snf2* gene
ICE*Ssu*YS205	*rplL*	IIIb	Vb	III	1	37.85	67,475	/	38.15	/	/	/	inside of *snf2* gene
ICE*Ssu*YS209	*rplL*	IIIb	Vb	III	1	37.58	61,552	/	37.61	/	/	/	inside of *snf2* gene
ICE*Ssu*YS388	*rplL*	IIIb	Vb	II	3	38.66	68,798	45.47	/	Serine3	pfam01076	3′ side of Peptidylprolyl isomerase	/
ICE*Ssu*YS430	*rplL*	IIIb	Vb	IVa	2	38.13	70,127	43.61	/	Serine3	pfam01076	inside of *snf2* gene	/
ICE*Ssu*YS538	*rplL*	IIIb	Vb	III	1	37.44	65,353	/	37.14	/	/	/	inside of *snf2* gene

/, Not available

### 2.2. Conjugation assay

The presence of the circular extrachromosomal form of ICEs in corresponding strains were firstly detected using specific primers facing outward from 5′ and 3′ side of each ICE (Supplementary Table S2). PCR amplification cycling parameters were 94°C for 5 min, then 30 cycles of 94°C for 30s, 48°C for 30s, 72°C for 60s. This was followed by a final elongation step at 72°C for 10 min. In mating experiment, *S. suis* derivative strain P1/7RIF (tetracycline-susceptible but rifampin resistant) was used as recipient and *S. suis* strains carrying circular ICEs (tetracycline-resistant but rifampin-susceptible) were utilized as donors. Donor and recipient strains were grown separately in THB broth containing the appropriate antibiotics at 5% CO_2_ 37°C until OD600 nm value reaching 0.6 in the logarithmic growth phase, and then 0.2 ml of donor culture was mixed with 1 ml of recipient culture. Strains were collected by centrifugation and were resuspended in 100 μl THB supplemented with 10 mM MgCl_2_, 2 μg/ml BSA, and 100 U Dnase I, then spread onto a sterile nitrocellulose membrane overlaid on a THB agar plate and incubated 6 h. Strains were removed by washing the filters in 1 ml of THB broth, then 100 μl of the solution was spread onto THB agar plate containing tetracycline (8 μg/ml) and THB agar plate containing both tetracycline (8 μg/ml) and rifampin (8 μg/ml), respectively. Transconjugants grown on the THB containing both tetracycline and rifampin were confirmed by PCR and sequencing. Conjugative transfer frequencies were calculated as the number of transconjugants/the number of strains grown on the THB containing tetracycline.

### 2.3. Nucleotide sequence accession numbers

The sequences of DNA elements obtained in this study were deposited in GenBank under accession numbers MK211778, MK211782–MK211826, MN394765, MN420460, and MK473142 (Supplementary Table S1).

## 3. Results

### 3.1. The distribution of AR genes in 155 *S. suis* genomes

One of 155 genomes did not harbor any AR genes. Totally, 656 AR genes were present in the remaining 154 genomes. The AR genes were clustered into 11 categories, consisting of tetracycline, macrolides, lincosamide, streptogramin, aminoglycoside, trimethoprim, amphenicols, nucleoside, quinupristin/dalfopristin, glycopeptide, and oxazolidinones resistance genes (Supplementary Table S1). It is noteworthy that 145 genomes harbored at least two categories of AR genes. Among them, 14ND95 and YS39 harbored 11 and 10 AR genes, respectively.

#### 3.1.1. The tetracyclines resistance genes

Totally, over 96% (150/155) of genomes carried 161 tetracycline-resistant genes, consisting of *tet* (O) (*n* = 102), *tet* (O/W/32/O) (*n* = 32), *tet* (M) (*n* = 12), *tet* (40) (*n* = 7), *tet* (W) (*n* = 4), *tet* (L) (*n* = 3), and *tet* (32) (*n* = 1) genes. Eleven of 150 genomes harbored two tetracycline-resistant genes simultaneously.

#### 3.1.2. The MLS, macrolides/streptogramin, and lincosamide resistance genes

Totally, 111 genomes carried 122 MLS genes, consisting of *erm* (A) (*n* = 11) and *erm* (B) (*n* = 111) genes. *Erm* (B) gene was prevalent and present in 104 genomes, while *erm* (A) gene was present in 11 genomes. The 35 macrolides/streptogramin resistance gene *msr* (D) (*n* = 15) and *mef* (A) (*n* = 20) was present in 20 genomes. Fifty-one lincosamide resistance genes were present in 33 genomes, consisting of *lnuB* (*n* = 17), *lnuC* (*n* = 4), *lnuD* (*n* = 12), *lsaE* (*n* = 14), *lsaC* (*n* = 3), and *lmrD* (*n* = 1) genes.

#### 3.1.3. The aminoglycosides resistance genes

In the present study, 60% (93/155) of carried 206 aminoglycosides resistance genes, consisting of *ant (6)-Ia* (*n* = 62), *aac (6′)-Ie-aph (2″)-Ia* (*n* = 50), *spw* (*n* = 39), *aph (3′)-IIIa* (*n* = 36), *ant (4′)-Ib* (*n* = 11), *aad* (*6*; *n* = 7), and *ant (4)-Ia* (*n* = 1) genes.

#### 3.1.4. The trimethoprim and chloramphenicol resistance genes

Three genomes harbored trimethoprim resistance gene *dfrF*. Fourteen genomes harbored chloramphenicol resistance gene, consisting of *cat* (*n* = 10), *cat-TC* (*n* = 3), and *cat-Q* (*n* = 1) genes.

In addition, 18 and one genomes harbored nucleoside resistance gene *sat-4* and quinupristin/dalfopristin-resistant gene *vatE*, respectively. It is noteworthy that YS191 harbored one glycopeptide resistance gene *vanXYG*.

### 3.2. Analysis of MGEs with ARs in 155 *S. suis* genomes

At *rplL* loci, 29 insertions were fully sequenced with no gap between the *rplL* and *hdy* genes. Nine of these 29 insertions lacked AR genes. Sequences of the remaining 20 insertions that contained AR genes were further analyzed. All of them harbored a 15-bp *att* sequence 5′-TTATTTAAGAGTAAC-3′ in the flanking region. The twenty insertions were composed of 18 ICEs, one DICESsuYS172 that lacked VirB4, and one tandem ICE*Ssu*YS19 (Table 1).

At *rum* loci, two intact ICEs with AR genes were identified and designated ICE*Ssu*YS64 and ICE*Ssu*14ND1 between the *rum* and *glf* genes (Table 1). ICE*Ssu*14ND1 harbored the 14-bp *att* sequences 5′-CACGTGGAGTGCGT-3′ and 5′-CACATAGAAGTTGT-3′ in the 5′ and 3′ side flanking regions, respectively. ICE*Ssu*YS64 harbored the 14-bp *att* sequences 5’-CACGTGGAGTGCGT-3′ and 5′ -CACGTTGAAGTTGT-3′ in the 5′ and 3′ side flanking regions, respectively. In the present study, ten intact prophages with AR genes were identified at *rum* loci (Supplementary Table S1). It is noteworthy that YS34 and YS162 harbored ICE and prophage simultaneously (Supplementary Table S1). Nine prophages with AR genes were integrated into downstream of *rum* gene and upstream of *pgmA* gene. One prophage (ΦSsuYS43) with AR genes was integrated into downstream of *rum* gene and upstream of *glf* gene.

In addition to the aforementioned MGEs present in 30 genomes (18 genomes carrying ICE, eight genomes carrying prophage, two genomes carrying both ICE and prophage, one genome carrying DICE, one genome carrying tandem ICE), 29 IMEs and 3 identical CIMEs with AR genes were found in remaining 125 genomes. The majority of AR-containing IMEs (27 out of 29) were integrated into the *snf2* gene (Table 2). The remaining two IMEs from genomes YS4 and YS67 were integrated into *ppi* loci (Table 2). It is noteworthy that 11 IMEs were integrated into the same contigs with either *rplL* or *hdy* genes, and the IME of genome YS12 was located in the same contig with the *glf* gene.

**Table 2:** Characteristics of IMEs and CIMEs identified in the present study.

IME name	Size (bp)	G + C (%)	Integrase of IME	Relaxase of IME	Integration site	IME_class
IME*Ssu*YS4	13,446	43.31	Serine3	pfam01076	*ppi*	IME_Class_6
IME*Ssu*YS12	11,016	46.06	Serine3	pfam01076	*snf2*	IME_Class_6
IME*Ssu*YS23	10,846	46.47	Serine3	pfam01076	*snf2*	IME_Class_6
IME*Ssu*YS44	13,185	43.69	Serine3	pfam01076	*snf2*	IME_Class_6
IME*Ssu*YS53	13,185	43.69	Serine3	pfam01076	*snf2*	IME_Class_6
IME*Ssu*YS67	10,955	45.76	Serine3	pfam01076	*ppi*	IME_Class_6
IME*Ssu*YS72	10,785	46.41	Serine3	pfam01076	*snf2*	IME_Class_6
IME*Ssu*YS77	10,984	46.41	Serine3	pfam01076	*snf2*	IME_Class_6
IME*Ssu*YS104	10,977	46.4	Serine3	pfam01076	*snf2*	IME_Class_6
IME*Ssu*YS106	10,983	46.15	Serine3	pfam01076	*snf2*	IME_Class_6
IME*Ssu*YS110	12,721	43.81	Serine3	pfam01076	*snf2*	IME_Class_6
IME*Ssu*YS111	12,649	44.07	Serine3	pfam01076	*snf2*	IME_Class_6
IME*Ssu*YS123	12,649	44.07	Serine3	pfam01076	*snf2*	IME_Class_6
IME*Ssu*YS124	12,649	44.07	Serine3	pfam01076	*snf2*	IME_Class_6
IME*Ssu*YS131	12,649	44.07	Serine3	pfam01076	*snf2*	IME_Class_6
IME*Ssu*YS163	10,977	46.59	Serine3	pfam01076	*snf2*	IME_Class_6
IME*Ssu*YS190	10,957	46.57	Serine3	pfam01076	*snf2*	IME_Class_6
IME*Ssu*YS201	10,981	46.52	Serine3	pfam01076	*snf2*	IME_Class_6
IME*Ssu*YS210	10,986	46.56	Serine3	pfam01076	*snf2*	IME_Class_6
IME*Ssu*YS249	10,986	46.57	Serine3	pfam01076	*snf2*	IME_Class_6
IME*Ssu*YS408	11,033	46.14	Serine3	pfam01076	*snf2*	IME_Class_6
IME*Ssu*YS488	10,983	47.56	Serine3	pfam01076	*snf2*	IME_Class_6
IME*Ssu*YS493	10,957	46.86	Serine3	pfam01076	*snf2*	IME_Class_6
IME*Ssu*YS498	10,957	46.86	Serine3	pfam01076	*snf2*	IME_Class_6
IME*Ssu*YS501	10,957	46.86	Serine3	pfam01076	*snf2*	IME_Class_6
IME*Ssu*YS525	13,530	45.21	Serine3	pfam01076	*snf2*	IME_Class_6
IME*Ssu*YS535	12,671	44.58	Serine3	pfam01076	*snf2*	IME_Class_6
IME*Ssu*YS543	12,671	44.58	Serine3	pfam01076	*snf2*	IME_Class_6
IME*Ssu*YS555	13,530	45.21	Serine3	pfam01076	*snf2*	IME_Class_6
CIME*Ssu*ND2	12,984	35.89	/	/	*snf2*	/
CIME*Ssu*14ND71	12,984	35.89	/	/	*snf2*	/
CIME*Ssu*14ND96	12,984	35.89	/	/	*snf2*	/

No insertions with ARs were identified at *mutT*, *luciferase-like monooxygenase*, and *ADP ribose pyrophosphatase* loci in the present study.

### 3.3. AR genes of MGEs

The 57 AR genes that were harbored in the 20 ICEs belonged to three categories: tetracycline, MLS, and aminoglycosides. Five ICEs carried all three categories of antibiotic resistance genes (Supplementary Table S1). The ICEs carried four types of tetracycline resistance genes, including *tet* (O/W/32/O), *tet* (O), *tet* (M), and *tet* (40) genes. The *tet* (O) gene was predominant and appeared in 11 ICEs. It is noteworthy that eight ICEs carried the *tet* (O/W/32/O) gene. The *tet* (40) and *tet* (M) genes were present in two and one ICEs, respectively (Supplementary Table S1). The *erm* (B) gene was found in 14 ICEs. Moreover, ICE*Ssu*14ND70 and ICE*Ssu*YS162 each carried three and two copies of the *erm* (B) gene, respectively (Supplementary Table S1). Eight ICEs that carried genes coding for aminoglycoside resistance, consisting of the *ant (6)-Ia* (*n* = 8), *spw* (*n* = 5), and *aph (3′)-IIIa* (*n* = 4) genes. Additionally, the *sat-4* gene that encodes for streptothricin resistance was found in ICE*Ssu*YS17.

Tandem ICE*Ssu*YS19 only carried the *tet* (O) gene, and the DICE*Ssu*YS172 carried both the *tet* (O) and *erm* (B) genes (Supplementary Table S1).

**Figure 1:**
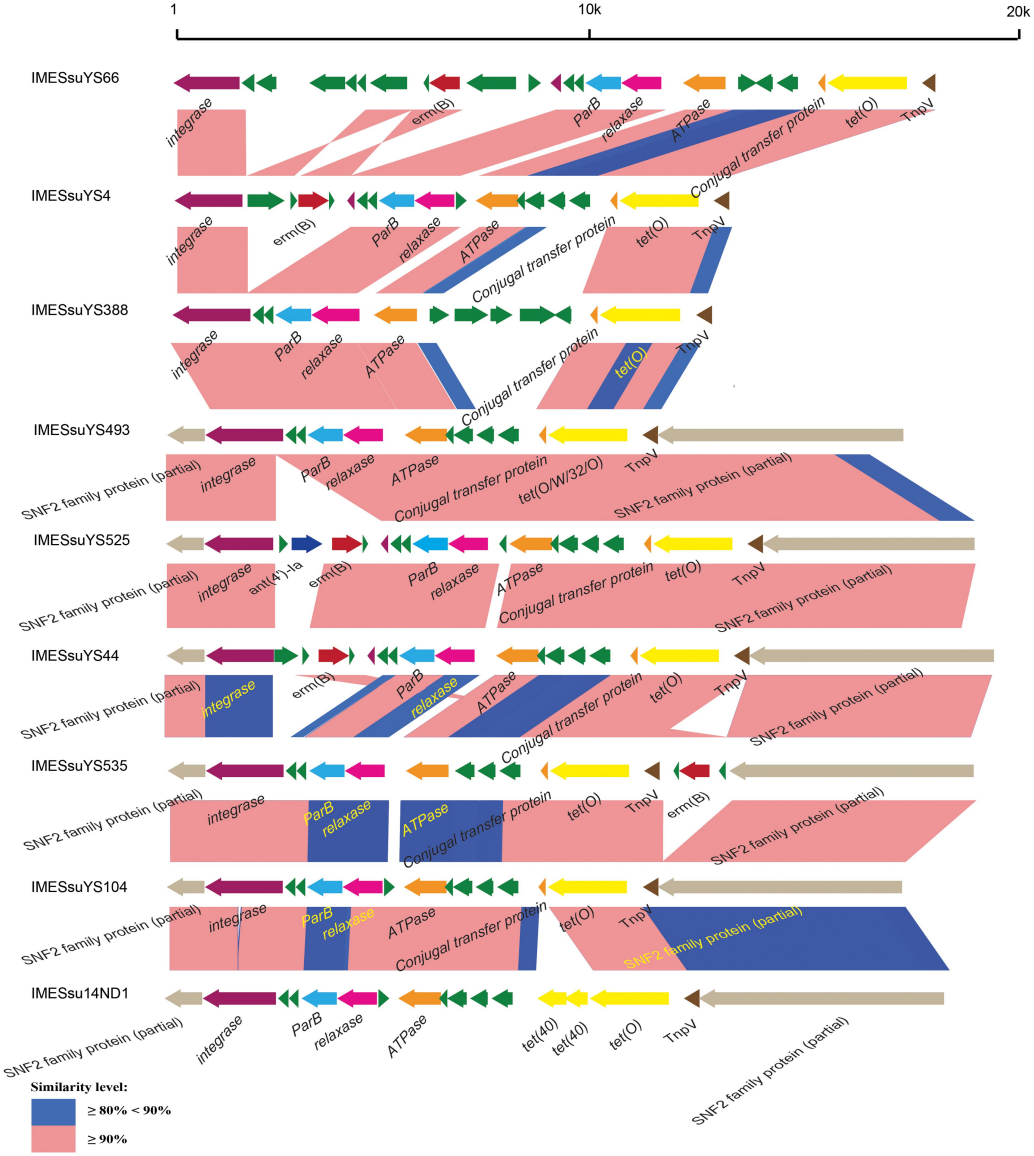
Schematic comparison of representative IMEs. The direction of the arrow indicates the direction of transcription. Regions of >90 and 80–90% identity are marked by pink and blue shading, respectively. The AR genes, *integrase* genes, *ParB* genes, *relaxase* genes, *ATPase* genes, *conjugal transfer* genes, *TnpV* genes, *snf2* genes are indicated by different colors.

The 43 AR genes present in 29 IMEs were mainly *tet* (O) and *erm* (B) genes. Among the 29 IMEs, 13 IMEs only carried the *tet* (O) gene. Ten IMEs carried both the *tet* (O) and *erm* (B) genes. Only two IMEs from genome YS525 and YS555 simultaneously carried *tet* (O), *erm* (B) and the aminoglycoside resistance gene *ant (4′)-ia* (Figure 1; Supplementary Table S1). Three identical CIMEs contained a combination of *aph (3′)-IIIa*, *aac (6′)-Ie-aph (2″)-Ia*, *ant (6)-Ia*, *tet* (O/W/32/O), *erm* (B), and *sat-4* genes (Supplementary Table S1).

The 38 AR genes harbored in the ten prophages belonged to five categories: tetracycline, macrolides, streptogramin, lincosamide, and aminoglycosides. The *tet* (O) and *erm* (B) gene was present in seven and four prophages, respectively. The prophages also carried *mef* (A) (*n* = 7), *msr* (D) (*n* = 5), *ant (6)-Ia* (*n* = 5), *aac (6′)-Ie-aph (2″)-Ia* (*n* = 4), *lnu*D (*n* = 2), *aph (3′)-IIIa* (*n* = 1), *lsaE* (*n* = 1), *lnu*B (*n* = 1), and *spw* (*n* = 1) genes (Supplementary Table S1).

### 3.4. Characterization of ICEs

**Figure 2:**
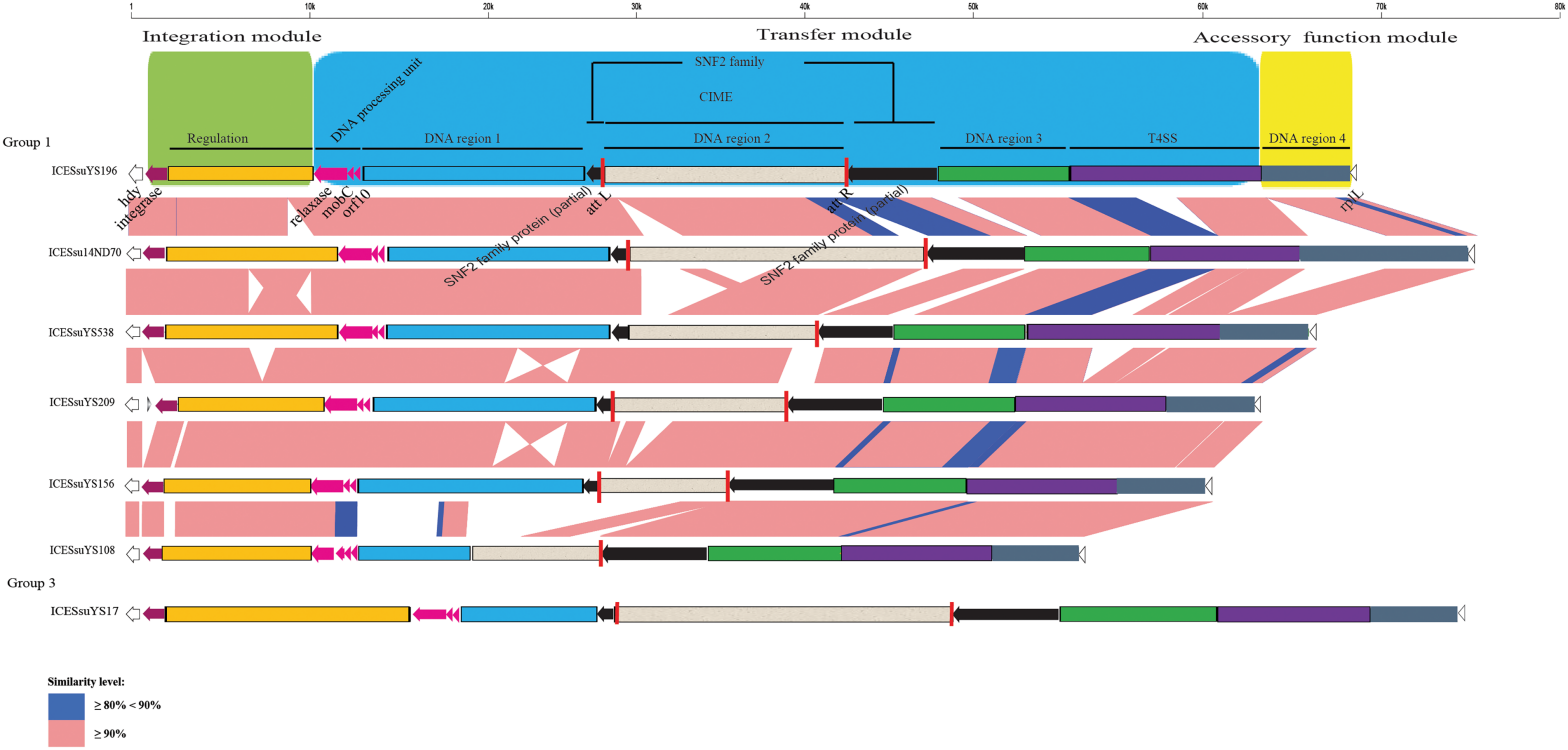
Schematic comparison of representative ICEs carrying CIMEs. The direction of the arrow indicates the direction of transcription. Regions of >90 and 80- 90% identity are marked by pink and blue shading, respectively. The DNA regions, T4SS, CIMEs, *integrase* gene, *relaxase* gene, *mobC*, *orf10*, *snf2* gene, *att* site, integration module, transfer module, and accessory module of ICEs are indicated by different colors. The *rplL* and *hdy* genes are located in the flanking region of the ICEs.

**Figure 3:**
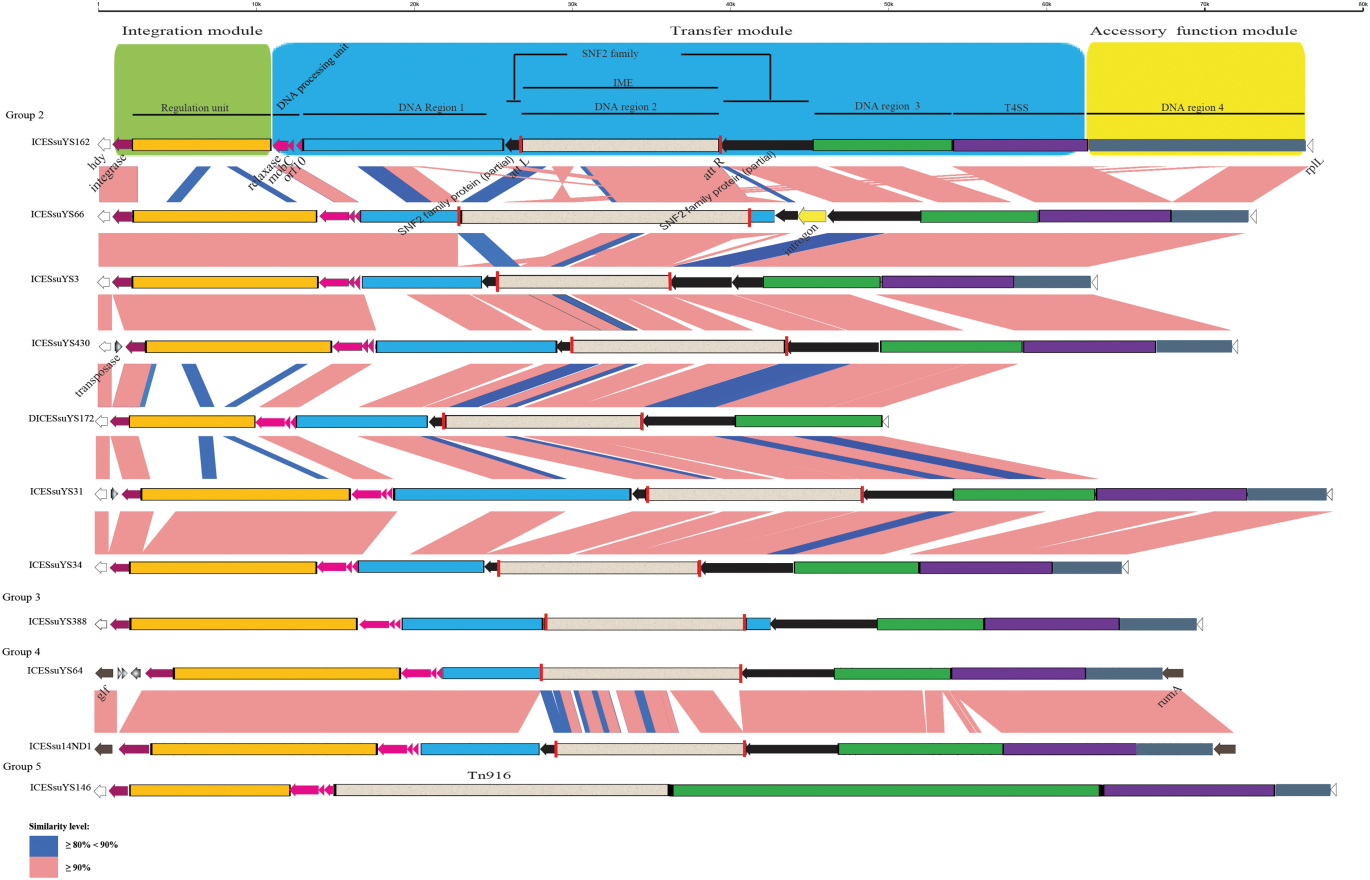
Schematic comparison of representative ICEs and DICEs carrying IMEs and transposon Tn*916*. The direction of the arrow indicates the direction of transcription. Regions of >90 and 80–90% identity are marked by pink and blue shading, respectively. The DNA regions, T4SS, IMEs, Tn*916*, *integrase* gene, *relaxase* gene, *mobC*, *orf10*, *snf2* gene, *att* site, integration module, transfer module, and accessory module of ICEs and DICEs are indicated by different colors. The *rplL* and *hdy* genes or *rumA* and *glf* genes are located in the flanking region of the ICEs and DICE.

We further investigated the characterizations of ICEs. In a previous study, the ICEs were classified into three groups (Tn*5252*, ICE*sp*1108, and TnGBS2) based on the presence of signature proteins VirB4, integrase, and relaxase (Huang et al., 2016a). The integrases of ICEs integrated into *rplL* and *rum* were tyrosine and serine integrase, respectively. The relaxases of all ICEs belonged to the MobP family. Moreover, all ICEs contained the conserved DNA processing unit composed of relaxase, MobC, and Tn*5252* ORF10 (Figures 2, 3).

All 20 ICEs belonged to the Tn*5252* group and ranged from 52,425 bp to 77,439 bp in size. Their G + C content ranged from 37.21 to 39.3%. Sequence alignment revealed high similarity between ICE*Ssu*YS10 and ICE*Ssu*YS430, ICE*Ssu*YS156 and ICE*Ssu*YS165, and ICE*Ssu*YS196 and ICE*Ssu*YS205, respectively. It is noteworthy that an intact type IV secretion system (T4SS) containing the *VirB1, VirB4, VirB6*, and *VirD4* genes was identified in the 20 ICEs.

There were five VirB4, integrase, and relaxase combinations in the 20 ICEs, primarily distinguished by variations in relaxase. The combinations were classified into five groups: clade III_b_ + clade V_b_ + clade III (group 1, *n* = 8), clade III_b_ + clade V_b_ + clade IV_a_ (group 2, *n* = 7), clade III_b_ + clade V_b_ + clade II (group 3, *n* = 2), clade III_b_ + clade III + clade II (group 4, *n* = 2) and clade III_b_ + clade V_b_ + clade IV_b_ (group 5, *n* = 1; Table 1).

The structures of all ICEs could be divided into an integration module, a transfer module, and an accessory function module. The integration module was composed of integrase and regulation unit, whereas the transfer module contained DNA processing unit, DNA region 1 to 3 and the T4SS. DNA region 4 constituted the accessory function module (Figures 2, 3).

Three modules were conserved within groups 1 and 4. Within groups 2 and 3, there were obvious variations in the integration and transfer modules. Variations were present mainly in regulation unit and DNA region 1 (Figures 2, 3).

The size and G + C content of DICE*Ssu*YS172 were 48,743 bp and 38.45%, respectively. The clades of integrase and relaxase in DICE*Ssu*YS172 were identical to those of group 2 ICEs (Table 1); However, an intact T4SS was absent. Compared with ICEs from other groups, the transfer module of DICE*Ssu*YS172 was most similar to that of group 2 ICEs (Figure 3).

The size and G + C content of the tandem ICE*Ssu*YS19 were 112,607 bp and 38.39%, respectively. Two clades of VirB4 (II + III_a_), integrase (IV + V_a_), and relaxase (I + IV_a_) were present in tandem ICE*Ssu*YS19, and it contained one copy of the T4SS (Table 1).

DNA region 2 of the ICEs contained various AR genes as DNA cargoes. In 15 ICEs, DICE*Ssu*YS172, and tandem ICE*Ssu*YS19, 17 cargoes were specifically integrated into the *snf2* gene, which encodes an SNF2 family protein. These cargoes harbored an 11-bp inverted repeat 5’-TTTTGCGGACA-3′ in the flanking region. Moreover, they also carried a *TnpV* transposase gene. It is noteworthy that ten of them possessed integrase and relaxase genes with a pattern similar to that of IMEs (Coluzzi et al., 2017; Figure 3; Table 1; Supplementary Figure S1). The IMEs were identified in eight ICEs, DICE*Ssu*YS172, and tandem ICE*Ssu*YS19. The remaining seven DNA cargoes that had lost the integrase and relaxase genes were designated *cis*-IMEs (CIMEs; Coluzzi et al., 2017; Figure 2; Table 1; Supplementary Figure S2).

IMEs were also found in ICE*Ssu*YS66 and ICE*Ssu*YS388, where they were inserted downstream of genes encoding peptidylprolyl isomerase (PPI; Libante et al., 2019; Figure 3; Table 1). ICE*Ssu*YS388 harbored an intact *snf2* gene, and a group II intron gene was integrated into the *snf2* gene of ICE*Ssu*YS66 (Table 1; Supplementary Figure S1). The two IMEs found in ICE*Ssu*YS66 and ICE*Ssu*YS388 harbored an inverted repeat sequence in the flanking region that was identical to that of the aforementioned ten IMEs. The integrases of aforementioned 12 IMEs were identical and belonged to serine integrase type 3 (Coluzzi et al., 2017), regardless of their integration site. Meanwhile, the relaxases of the IMEs belonged to the PF01076/MOBV superfamily. Based on their integrase and relaxase types, all 12 IMEs belonged to IME_Class_6 (Coluzzi et al., 2017). Interestingly, the G + C content of the IMEs (from 39.69 to 47.65%) was clearly different from that of the corresponding ICEs (from 37.21 to 39.3%; Table 1).

CIMEs were also found in ICE*Ssu*YS108 and ICE*Ssu*YS538, in which the 3′ side of the *snf2* gene (for ICE*Ssu*YS108) and the *attL* site (for ICE*Ssu*YS538 and ICE*Ssu*YS108) were absent (Figure 2; Supplementary Figure S2). On the contrary, the G + C content of the all CIMEs integrated into ICEs were similar to those of their host ICEs, except that of CIME harbored by ICE*Ssu*YS108 was unavailable. Interestingly, all ICEs from group 1 carried CIMEs (Table 1).

Transposon Tn*916*, prevalent in the epidemic strains that caused *S. suis* outbreak in China 2005 (Ye et al., 2008), was integrated into ICE*Ssu*YS146 (Figure 3; Supplementary Figure S2).

All AR genes in the ICEs were carried by integrated DNA cargos, except for an additional *erm* (B) gene that was carried in the accessory function module of ICE*Ssu*YS162 and ICE*Ssu*14ND70. Unlike IMEs and CIMEs, transposon Tn*916* only harbored the *tet* (M) gene. Compared with IMEs that only carry the *erm* (B) gene and/or tetracycline resistance gene, aminoglycoside resistance genes were wildly present in nine CIMEs.

Moreover, additional 29 IMEs with AR genes present in remaining genomes also belonged to Class_6 IMEs. It is noteworthy that their G + C content ranged from 43.31 to 47.56% that were similar to those of IMEs integrated into ICEs (Table 2).

The size and G + C content of prophages ranged from 58,465 bp to 87,220 bp and from 38.83 to 39.61%, respectively. Among ten prophages, five prophages ΦSsuYS199, ΦSsuYS214, ΦSsuYS225, ΦSsuYS255 and ΦSsuYS262 were completely identical.

### 3.5. Transferability of ICEs

The presence of the circular extrachromosomal form of 18 ICEs at *rpIL* loci were detected in corresponding strains. The circularization of eight ICEs were detected in corresponding strains, consisting of ICE*Ssu*YS3, ICE*Ssu*YS17, ICE*Ssu*YS34, ICE*Ssu*YS66, ICE*Ssu*YS146, ICE*Ssu*YS162, ICE*Ssu*YS388, and ICE*Ssu*YS430. Notable, none of circular ICE carried CIMEs was detected in corresponding strain. In conjugation assay, eight *S. suis* strains carrying circular ICEs were used as donors and *S. suis* strain P1/7RIF were utilized as recipients. In mating experiments, we obtained clones of transconjugant carrying ICE*Ssu*YS3, ICE*Ssu*YS34, and ICE*Ssu*YS388 in *S. suis* P1/7RIF at a low frequency of ∼7.8 × 10^−8^ (3/3.84 × 10^7^), 1.2 × 10^−7^ (4/3.26 × 10^7^), and 7.1 × 10^−8^ (2/2.81 × 10^7^), respectively.

## 4. Discussion

*S. suis* strains from swine are important reservoirs of antibiotic resistance genes in that tetracycline, lincosamide, macrolide, and aminoglicoside are extensively used for therapy and metaphylaxis in swine industry (Schwarz et al., 2007; Callens et al., 2012; Seitz et al., 2016; Xu et al., 2017). In the present study, 155 *S. suis* genomes harbored 656 AR genes which were mainly clustered into tetracycline, macrolide, lincosamide, and the aminoglycosides resistance genes. In the present study, 154 of 656 AR genes were present in MGEs, consisting of 20 ICEs, one tandem ICE, one DICE, 29 IMEs, three CIMEs, and ten prophages. Interestingly, the AR genes in MGEs were also responsible for the resistance to the aforementioned antibiotics in the present study. The MGEs played significant roles in the acquisition and dissemination of AR genes in *S. suis*. Interestingly, these resistance genes carried by MGEs also emerged and were prevalent in *S. suis* strains from patients in China (Wang et al., 2019), which is becoming emergent threat to local public health.

Consistent with previous report, ICEs and IMEs were prevalent vehicles of AR genes in *S. suis* (Huang et al., 2016a; Libante et al., 2019; Liang et al., 2021). The ICEs are able to excise from the chromosomes to form circular intermediates prior to conjugative transfer into recipient cells (Burrus et al., 2002). In the present study, eight of 20 ICEs did excise from the corresponding chromosome to produce a circular form, which represented an intermediate required for their transference. In addition, clones of transconjugant carrying three ICEs (ICE*Ssu*YS3, ICE*Ssu*YS34, and ICE*Ssu*YS388) were obtained in mating experiment. Our results indicated that the ICEs, as mobile genetic elements, may be crucial for the acquisition and dissemination of AR genes in *S. suis*.

Integrase, relaxase, and VirB4 as signature proteins of integration and transfer modules were used to classify the ICEs. The predominant clades of VirB4 and integrase in the Tn*5252* group are Vb and IIIb (Huang et al., 2016a), consistent with what we have observed in the current study. Substantial diversity was found in relaxase, which was classified into four clades. Relaxase clade III and IVa were predominant in the present study, while clade IVb was prevalent in the Tn*5252* group (Huang et al., 2016a). Five VirB4, integrase, and relaxase combinations were found in the 20 ICEs.

In the present study, three types of DNA cargo with AR genes were integrated into 20 ICEs, consisting of IMEs, CIMEs, and the transposon Tn*916*. It is noteworthy that nearly all AR genes in ICEs were carried by these three types of DNA cargoes. Therefore, it is reasonable to speculate that these cargoes play critical roles in the dissemination of AR genes. The three types of cargo contained different categories of AR genes. IMEs mainly harbored tetracycline and MLS resistance genes. CIMEs frequently contained genes coding for aminoglycoside resistance, while transposon Tn*916* carried only the *tet* (M) gene. In contrary to IMEs mainly carrying the *tet* (O) gene, the predominant tetracycline resistance gene in CIMEs was *tet* (O/W/O/32).

The IMEs could excise and integrate but carry only some of the sequences or genes necessary for their conjugative transfer. Instead, they employ the conjugative elements of co-resident ICEs to affect transfer. In our study, several lines of evidence indicated that IMEs were exogenous and transferable, even though IMEs were unable to excise in laboratory experiment (Libante et al., 2019, (1) their G + C content was obviously different from that of corresponding ICEs, (2) they had an insertion hot spot, (3) they had an 11-bp inverted repeat sequence in the flanking region as *att* site, (4) spontaneous excision may occur through their common site-specific integrase gene, (5) they carried a *TnpV* transposase gene, which has been widely reported to be involved in conjugative element integration (Wang et al., 2006; Huang et al., 2016b), and (6) they were widely distributed in strains from different origins. In the present study, IMEs were distributed in ten ICEs, one DICE, one tandem ICE, and 29 additional genomes. Similar IMEs were also integrated into *snf2* gene of ICE*Ssu*ZJ20091101-1 (KX077882.1), ICE*Ssu*LP081102 (KX077885.1), ICE*Ssu*JH1301 (KX077887.1; Huang et al., 2016a) and ICE*SsD9* (Huang et al., 2016b). Through site-specific recombination mechanisms, IMEs integrated into ICEs/genomes and conferred antibiotic resistance to host ICEs/genomes. It is likely that the exchange, acquisition, and deletion of the IME module contributed to the evolution of ICEs.

IMEs encode tyrosine integrase, serine integrase, or DDE transposase. Tyrosine integrases were detected in more than 89% of *Streptococcus* IMEs (Coluzzi et al., 2017). In our study, all IMEs harbored the same serine integrase, Ser_3, which confers high specificity of IME integration. IME relaxases are classified into nine superfamilies, and the relaxases in this study belonged to the Rel_PF01076/MobV superfamily. In contrast to data from a previous study in which IMEs from classes 1, 2, 3, 4, 7, and 8 were present in 120 of 144 *Streptococcus* species (Coluzzi et al., 2017), all IMEs we analyzed in the present study were IME_class_6. IMEs were reported to be more widespread than ICEs in *S. suis* (Libante et al., 2019; Liang et al., 2021). In the present study, IMEs were also found to be highly prevalent in tested *S. suis* genomes. We propose that IMEs may play a critical role in the spread of tetracycline, macrolide, and lincosamide resistance genes and that their activity may explain the high rate of resistance to these antibiotics in the *S. suis* population.

CIMEs are decayed IMEs, which are *cis*-mobilizable elements that had lost their integration and relaxase genes but retained their *attL* and *attR* sites. CIMEs were found in nine ICEs and three additional genomes. A similar CIME was also inserted into the same integration site of ICE*Ssu*BSB6 (Supplementary Figure S2; Huang et al., 2018). The structure of the CIMEs was homologous to that of the mobile 15 K element of ICE*Ssu*32457 obtained from an Italian strain, although the latter had 1.3 kb *att* sequences in the flanking region and was integrated between the intact *snf2* gene and the DNA primase gene (Palmieri et al., 2012; Supplementary Figure S1). Despite the lack of a recombinase gene, the 15 K element may be capable of spontaneous excision *via* its 1.3 kb *att* sequences in the flanking region (Palmieri et al., 2012). Unlike IMEs, CIMEs frequently contained genes coding for resistance to aminoglycosides. We suggest that CIMEs may play an important role in the dissemination of aminoglycoside resistance. Further study is needed to investigate whether CIMEs can be excised by their own 11-bp *att* sequences in the flanking region and whether the transfer can be mediated by the *TnpV* gene.

In previous study, most of the IMEs carrying AR genes were integrated into *snf2* or *ppi loci* (Libante et al., 2019; Liang et al., 2021). In the present study, IMEs and CIMEs were predominantly inserted into *snf2* site. Moreover, a group II intron was site-specifically integrated into the *snf2* gene of ICE*Ssu*YS66. It is unclear why the *snf2* gene was the preferred integration site for these cargoes. The *snf2* gene was extensively distributed in the 155 *S. suis* genomes, only eight of which carried an intact *snf2* gene (data not shown). We proposed that a variety of cargos specifically integrated into the *snf2* gene may confer significant selective advantages to their host.

ICE*Ssu*YS146 harbored the transposon Tn*916*, which contributes to the spread of the *tet* (M) gene. It is noteworthy that *tet* (M) is a prevalent tetracycline resistance gene in *S. suis* strains from meningitis patients in Vietnam and outbreak in China, where it is also associated with the presence of transposon Tn*916* (Ye et al., 2008; Hoa et al., 2011). The capacity of transposon Tn*916* with the *tet* (M) gene to horizontal transfer in *S. suis* strains was revealed and played important roles in the evolution of the epidemic *S. suis* ST7 clone in our previous study (Ye et al., 2008).

The mobility of several ICEs was also evaluated in the present study. In *S. suis*, the ICEs carrying AR genes transferred in intra- and interspecies with frequency from 10^−3^ to 10^−8^ (Li et al., 2011; Marini et al., 2015; Huang et al., 2016a,b; Libante et al., 2019; Pan et al., 2019; Shang et al., 2019; Yang et al., 2022). The formation of circular intermediates between the *attL* and *attR* sites with the aid of the integrase was a requisite for the conjugative transfer of the ICEs (Flannery et al., 2011; Li et al., 2011). In the present study, the circular intermediates of eight ICEs were detected in corresponding strains, which were from group 2, 3, and 5. Notable, ICEs from group 1 did not appear to excise, even though they carried same clade of integrase to those of ICEs from group 2, 3, and 5. It is noteworthy that CIMEs mainly harbored aminoglycoside resistance genes were integrated into all ICEs from group 1. We proposed that multiple mutations in integrase genes and the integration of CIMEs may impair the excision and transferability capacity of ICEs from group 1.

In conjugation assay, the effective conjugation was not observed in five of excised ICEs *in vitro*. Similar observations were also reported in ICE*Ssu*HN105 (Zhu et al., 2019) and ICE*Ssu*NSU1086 (Libante et al., 2019). Moreover, clones of transconjugant carrying ICE*Ssu*YS3, ICE*Ssu*YS34, and ICE*Ssu*YS388 were obtained at a low frequency. Relaxase and T4SS components played critical roles in the transferability of ICEs (Li et al., 2011). Truncated *virB4* and *relaxase* gene was present in ICE*Ssu*YS146 and ICE*Ssu*YS162, respectively. The mutations and truncation of relaxase and T4SS components genes may contribute to the transfer deficiencies of corresponding ICEs. It is possible that the conjugative frequency of these ICEs below the detection limit or their self-transmission occurred only in specific conditions not met *in vitro*. Previous study reported that IME was not mobile anymore as a single element but was transferred passively by the ICE harbored it (Libante et al., 2019). Further studies are needed to investigation the excision and conjugation of the IMEs.

In the present study, ten prophage carrying AR genes were also detected at *rum* loci. ΦSsuYS34 and ΦSsuYS771 closely resembled ΦSsUD.1 (Accession No. FN677480) of *S. suis* (Palmieri et al., 2011a). ΦSsuYS199, ΦSsuYS214, ΦSsuYS225, ΦSsuYS255 and ΦSsuYS262 were identical and closely resembled Φ46.1 (Accession No. FM864213) of *S. pyogenes* (Brenciani et al., 2010). ΦSsuYS43 closely resembled ΦSsuHCJ3 of *S. suis* (Accession No. MN270269). The prophage was reported to be the vehicle of tetracycline, macrolide, lincomycin, amphenicols, oxazolidinones, and aminoglycoside resistance genes in *S. suis* strains (Wang et al., 2021; Uruen et al., 2022). The transfer of prophage is mainly through transduction by three mechanisms, called generalized, specialized, and lateral transduction. There is *in vitro* evidence of transfer of prophage Φm46.1 between *S. suis* and *S. pyogenes* in both directions with different transfer rate (from *S. suis* to *S. pyogenes* with 8.5 × 10^−4^ and from *S. pyogenes* to *S. suis* with 2.3 × 10^−9^; Giovanetti et al., 2014). Further studies are needed to investigate the transferability of the prophage identified in the present study.

Most of clinical *S. suis* strains remain sensitive to beta-lactams antibiotic, including penicillin and cephalosporin (Hoa et al., 2011; Wang et al., 2019; Bamphensin et al., 2021). Resistance to penicillin in *S. suis* strains isolated from pigs is gradually increasing (Zhang et al., 2008; Lunha et al., 2022). In the present study, *S. suis* strains YS162 and YS388 were resistance to penicillin with the minimum inhibitory concentration (MIC) value 6 μg/ml for each strain, meanwhile *S. suis* strain YS34 was resistant to amoxicillin with MIC value 64 μg/ml. Moreover, *S. suis* strains 14ND70, YS34, and YS388 were resistant to cefaclor with MIC value 32 μg/ml, 6 μg/ml, and 8 μg/ml, respectively. In addition, *S. suis* strain YS34 was also resistant to cefepime, cefotaxime, ceftriaxone, and cefuroxime with MIC value 12 μg/ml, 16 μg/ml, 24 μg/ml and 24 μg/ml, respectively. In *streptococci*, variations of penicillin-binding proteins (PBP) were essential for the beta-lactam resistance (Sauvage et al., 2008; Li et al., 2016). Five *pbp* genes have been identified in *S. suis* strains, *pbp1a*, *pbp1b*, *pbp2a*, *pbp2b*, and *pbp2x*. It is noteworthy that none of *pbp* genes were present in ICEs, IME, and prophage identified in the present study. Compared to those of *S. suis* P1/7 (penicillin-susceptible isolate; *pbp1a*: SSU0370, *pbp1b*: SSU0121, *pbp2a*: SSU1777, *pbp2b*: SSU1186, and *pbp2x*: SSU1548,), multiple nucleotide substitutions were present in *pbp* genes of aforementioned beta-lactams resistant strains (data were not shown). Further studies are needed to corelated the *pbp* gene mutations to the reduction of drug affinity.

An obvious drawback to the study was the use of draft genomes, which may have caused some limitations. Specifically, some ICEs and IMEs containing AR genes may not have been found. In addition, different classes of IMEs were not comprehensively identified. As a result, the prevalence, diversity, and contribution of ICEs and IMEs to the dissemination of AR genes may have been underestimated in the *S. suis* population.

In conclusion, AR-associated ICEs, DICEs, and a tandem ICE in *S. suis* strains from 155 genomes were identified and characterized. A particularly interesting finding was that the most common IMEs and CIMEs integrated into the ICEs and genomes in a site-specific manner. Our findings provide novel insights into the transmission patterns of AR genes and the evolutionary mechanisms of ICEs in *S. suis*.

## Data availability statement

The datasets presented in this study can be found in online repositories. The names of the repository/repositories and accession number(s) can be found in the article/Supplementary material.

## Author contributions

HZ, ZW, and JX designed the project. HZ drafted the manuscript. JW and XB carried out the experiments and generated the data. KQ, WK, PL, and HZ analyzed the data. All authors contributed to the article and approved the submitted version.

## Funding

This work was supported by the Priority Project on Infectious Disease Control and Prevention from the Ministry of Science and Technology of the People’s Republic of China (grant no. 2017ZX10303405-002, 2018ZX10734404) and the National Natural Science Foundation of China (grant no. 81572044).

## Conflict of interest

The authors declare that the research was conducted in the absence of any commercial or financial relationships that could be construed as a potential conflict of interest.

## Publisher’s note

All claims expressed in this article are solely those of the authors and do not necessarily represent those of their affiliated organizations, or those of the publisher, the editors and the reviewers. Any product that may be evaluated in this article, or claim that may be made by its manufacturer, is not guaranteed or endorsed by the publisher.

## Footnotes

^1^ https://cge.Food.dtu.dk/services/ResFinder/

^2^ https://github.com/dupengcheng/BlastViewer

## Supplementary material

SUPPLEMENTARY FIGURE 1 Schematic comparison of ICESsu32457 carrying 15K fragment and representative ICEs carrying IMEs. The direction of the arrow indicates the direction of transcription. Regions of >90% and 80%-90% identity are marked by pink and blue shading, respectively. The integrase genes, relaxase genes, snf2 genes, ppi genes, AR genes, and T4SS of ICEs are indicated by different colors. IMEs and the 15K fragment are highlighted in a red box. The AR genes and Tnpv genes of IMEs and the 15K fragment are indicated by different colors. The site-specific integrase, AR genes and Tnpv genes of IMEs are indicated by different colors. The rplL and hdy genes are located in the flanking regions of the ICEs.

SUPPLEMENTARY FIGURE 2 Schematic comparison of ICESsuBSB6 carrying 15K fragment and representative ICEs carrying CIMEs or Tn916. The direction of the arrow indicates the direction of transcription. Regions of >90% and 80%-90% identity are marked by pink and blue shading, respectively. The integrase gene, relaxase gene, snf2 gene, AR genes, and T4SS of ICEs are indicated by different colors. CIMEs, Tn916, and the 15K fragment are highlighted in a red box. The AR genes and Tnpv genes of CIMEs and the 15K fragment are indicated by different colors. The rplL and hdy genes are located in the flanking regions of the ICEs.

SUPPLEMENTARY TABLE 1 The information of the genomes and AR genes carried in the present study.

SUPPLEMENTARY TABLE 2 The primers used in the present study for the circular ICEs analyses.
